# LIGHT/TNFSF14 increases osteoclastogenesis and decreases osteoblastogenesis in multiple myeloma-bone disease

**DOI:** 10.18632/oncotarget.2633

**Published:** 2014-12-19

**Authors:** Giacomina Brunetti, Rita Rizzi, Angela Oranger, Isabella Gigante, Giorgio Mori, Grazia Taurino, Teresa Mongelli, Graziana Colaianni, Adriana Di Benedetto, Roberto Tamma, Giuseppe Ingravallo, Anna Napoli, Maria Felicia Faienza, Anna Mestice, Paola Curci, Giorgina Specchia, Silvia Colucci, Maria Grano

**Affiliations:** ^1^ Department of Basic and Medical Sciences, Neurosciences and Sense Organs, section of Human Anatomy and Histology, University of Bari, Bari, Italy; ^2^ Department of Emergency and Organ Transplantation, Section of Hematology with Transplantation, University of Bari, Bari, Italy; ^3^ Department of Clinical and Experimental Medicine, University of Foggia, Foggia, Italy; ^4^ Department of Emergency and Organ Transplantation, University of Bari, Bari, Italy; ^5^ Department of Biomedical Sciences and Human Oncology, University of Bari, Bari, Italy

**Keywords:** LIGHT/TNFSF14, multiple myeloma, bone disease, osteoclast, osteoblast

## Abstract

LIGHT, a TNF superfamily member, is involved in T-cell homeostasis and erosive bone disease associated with rheumatoid arthritis. Herein, we investigated whether LIGHT has a role in Multiple Myeloma (MM)-bone disease. We found that LIGHT was overproduced by CD14+ monocytes, CD8+ T-cells and neutrophils of peripheral blood and bone marrow (BM) from MM-bone disease patients. We also found that LIGHT induced osteoclastogenesis and inhibited osteoblastogenesis. In cultures from healthy-donors, LIGHT induced osteoclastogenesis in RANKL-dependent and -independent manners. In the presence of a sub-optimal RANKL concentration, LIGHT and RANKL synergically stimulated osteoclast formation, through the phosphorylation of Akt, NFκB and JNK pathways. In cultures of BM samples from patients with bone disease, LIGHT inhibited the formation of CFU-F and CFU-OB as well as the expression of osteoblastic markers including collagen-I, osteocalcin and bone sialoprotein-II. LIGHT indirectly inhibited osteoblastogenesis in part through sclerostin expressed by monocytes. In conclusion, our findings for the first time provide evidence for a role of LIGHT in MM-bone disease development.

## INTRODUCTION

Multiple Myeloma (MM)-bone disease, characterized by osteolytic lesions, is the most frequent clinical manifestation of symptomatic MM, being detected in 70 to 80% of patients at diagnosis and up to 90% at relapse. It increases the risk of skeletal-related events such as bone pain, pathological fractures, and spinal cord compression [1].

Osteolytic lesions result from an imbalance between increased osteoclast (OC) activity and reduced osteoblast (OB) repair [2–4]. The latter has also been related to suppressed functions of Wnt-signalling due to MM-cells through the expression of Wnt inhibitors such as dickkopf-1 (DKK1) and Sclerostin [5–11]. In addition, several cytokines belonging to tumour necrosis factor superfamily (TNFSF) have been implicated in the increased osteoclastogenesis [2, 3, 12–13]. Among these, decoy receptor 3 (DcR3) plays an important role in the OC formation occurring in MM-bone disease, as we previously described [14]. DcR3 is known to be also a soluble receptor of LIGHT [15] (homologous to *L*ymphotoxins exhibiting Inducible expression and competing with herpes simplex virus *G*lycoprotein D for herpes virus entry mediator [*H*VEM], a receptor expressed by *T* lymphocytes), whose potential involvement in MM is unknown.

LIGHT is a member of TNFSF (TNFSF14) expressed on cells with an immunological role such as activated T-cells, monocytes, granulocytes, spleen cells, and immature dendritic cells [15, 16]. As membrane-anchored or secreted form, LIGHT can bind two membrane-bound TNFSF signalling receptors, HVEM and lymphotoxin beta receptor (LTβR). HVEM is expressed on endothelial, dendritic, natural killer, T- and B-cells [17, 18] while LTβR is expressed on fibroblasts, monocytes, endothelial, epithelial and stromal cells [19]. Following the interaction of LIGHT with HVEM or LTβR, the recruitment of TNF receptor (TNFR)-associated factor-2 (TRAF2) and TRAF5 occurs, leading to gene induction through the activation of Nuclear-Factor-kappaB (NFκB) or c-Jun N-terminal kinase (JNK)/ activator protein 1 (AP-1) pathway, and finally resulting in cytokine production, cell survival or proliferation [20–23]. The LIGHT–LTβR interaction can also lead to cell death through the recruitment of TRAF3 and subsequent activation of caspases [24, 25]. Through the interaction with HVEM, LIGHT is described as a potent T-cell co-stimulatory molecule [13, 17, 26, 27]; its constitutive expression on T-cells causes activation and expansion of these cells, favouring the development of autoimmune diseases [28, 29]. Moreover, LIGHT has been implicated in rheumatoid arthritis bone erosions [30, 31]. To date, there are three literature reports on the contribution of LIGHT to OC formation, reaching conflicting results [30–32]. In particular, LIGHT was reported to induce *in vitro* differentiation of OCs from peripheral blood (PB) CD14+ monocytes of healthy-donors, when co-cultured with nurse-like cells isolated from the synovium of patients with rheumatoid arthritis [30]. Conversely, no OCs differentiated from the same CD14+ monocytes cultured alone [30]. In addition, other Authors reported that, in the presence or absence of the key pro-osteoclastogenic cytokine receptor activator of nuclear factor-kappaB ligand (RANKL), LIGHT induced OC differentiation from human peripheral blood mononuclear cells (PBMCs) of healthy-donors [31, 32]. The *in vitro* data regarding the LIGHT pro-osteoclastogenic role as well as the LIGHT high serum levels [31] found in rheumatoid arthritis patients supported a LIGHT contribution to the pathological bone resorption.

Based on the above literature data and consistently with our previous studies [8, 12, 14], we investigated the expression of LIGHT in MM patients and the role that this cytokine may play in the osteoclastogenesis and osteoblastogenesis occurring in MM-bone disease.

## RESULTS

### LIGHT expression in monocytes, T-cells, neutrophils and myeloma-cells from patients and controls

**Figure 1:**
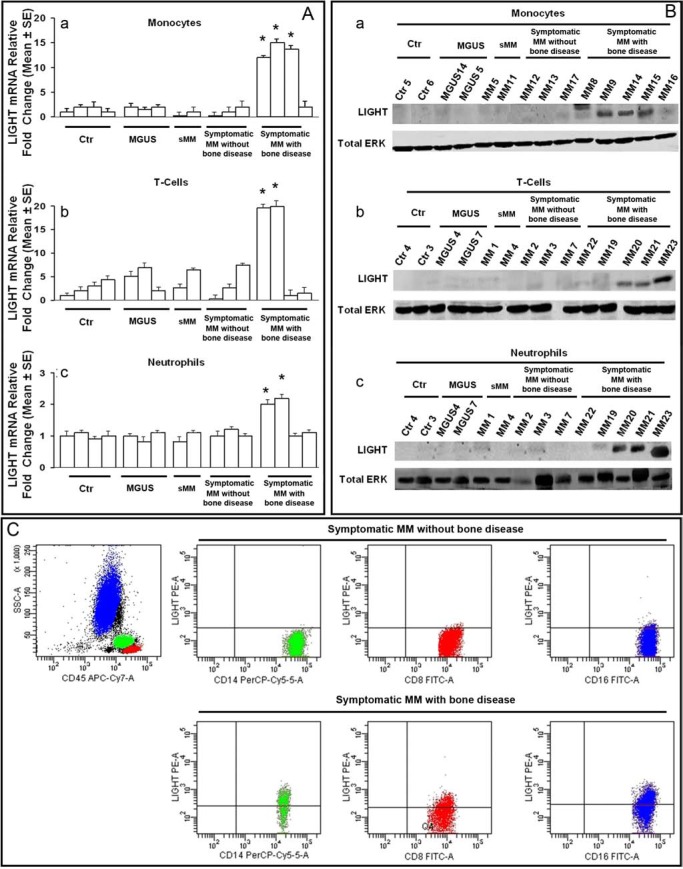

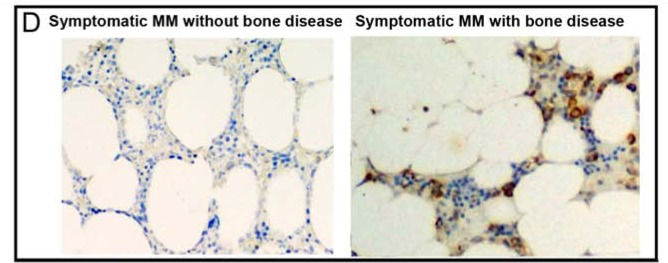
LIGHT expression in patients and controls Monocytes, T-cells and neutrophils from peripheral blood of controls (ctr), MGUS, smoldering MM (sMM) and symptomatic MM patients without or with bone disease were assessed for LIGHT expression by real-time PCR **(A)**, western blotting **(B)** and flow cytometry **(C)**. LIGHT immunostaining was performed in bone marrow biopsies **(D)**. The lowest mRNA levels of LIGHT were detected in monocytes (Aa), T-cells (Ab) and neutrophils (Ac) from controls, MGUS, sMM and symptomatic MM patients without bone disease. As compared with all of them, LIGHT mRNA higher levels of 11-fold (*P* < 0.0001) in monocytes (Aa), 9.2 fold ( *p* < 0.001) in T-cells (Ab) and 1.8- fold ( *p* < 0.01) in neutrophils (Ac) were detected in symptomatic MM patients with bone disease. In these latter, high levels of LIGHT protein were detected by western blotting in 90% of monocytes (Ba), 50% of T-cells (Bb) and 50% of neutrophils (Bc), whereas the samples of symptomatic MM patients without bone disease, sMM, MGUS and controls did not display a detectable LIGHT protein amount. Flow cytometry dot plots showed LIGHT expression on CD14+ monocytes, CD8+ T-cells and CD16+ neutrophils from a representative symptomatic MM patient without bone disease, and a symptomatic MM patient with bone-disease; in particular, LIGHT levels were higher in the former than in the latter (C). LIGHT positive immunostaining was also observed in the bone marrow biopsy from a representative symptomatic MM patient with bone-disease; conversely, LIGHT resulted negative in a symptomatic MM patient without bone-disease (D). The photomicrographs were obtained using a Nikon Eclipse E400 microscope equipped with a Nikon plan Apo 20×/0.75 DICM (Nikon, Italia), Magnification 200X. ERK, extracellular signal-regulated kinase.

By means of real-time PCR, western blotting, flow cytometry and immunohistochemistry, we assessed the expression of LIGHT in BM aspirates and biopsies from patients as well as in PB from patients and healthy-donors. Using these different methods, LIGHT resulted overexpressed in 52/58 (90%) of MM-bone disease samples, at both mRNA and protein levels; otherwise in all the other samples, its expression resulted at the lowest detectable levels by real-time PCR, and undetectable by western blotting. In particular, LIGHT expression was detected in CD14^+^ monocytes from all the positive samples whereas, in 50% of them, it was detected in CD2^+^ T-cells and/or neutrophils, too. The above results, referred to PB samples analyzed by real-time PCR and western blotting, are shown in Figures 1A and 1B, respectively. The corresponding BM samples gave overlapping results (data not shown). In Table 1, the mean values of the flow cytometry results are detailed; they are referred to CD14^+^ monocytes, CD16^+^ neutrophils and CD8^+^ T-cells. The latter cells were identified as the main LIGHT expressing T-cell subset in MM-bone disease samples. Representative dot plots of LIGHT cell expression are shown in Figure 1C.

**Table 1:** Cytofluorimetric expression of LIGHT in CD14+ Monocytes, CD8+ T-cells and CD16+ Neutrophils from all peripheral blood and bone marrow samples

Peripheral Blood								
CD14+ Monocytes			CD8+ T-cells			CD16+ Neutrophils		
Healthydonors	Symptomatic MM w/o bone disease	Symptomatic MM with bone disease	Healthy-donors	Symptomatic MM w/o bone disease	Symptomatic MM with bone disease	Healthy-donors	Symptomatic MM w/o bone disease	Symptomatic MM with bone disease
1 ± 0.5	3.6 ± 2.8	47.1 ± 9.5*	1.1 ± 0.5	1 ± 0.5	8.0 ± 5.5§	1 ± 0.9	2 ± 1.1	40.3 ± 17.8*
**Bone Marrow**								
**CD14+ Monocytes**			**CD8+ T-cells**			**CD16+ Neutrophils**		
**Patients with non-neoplastic disease**	**Symptomatic MM w/o bone disease**	**Symptomatic MM with bone disease**	**Patients with non-neoplastic disease**	**Symptomatic MM w/o bone disease**	**Symptomatic MM with bone disease**	**Patients with non-neoplastic disease**	**Symptomatic MM w/o bone disease**	**Symptomatic MM with bone disease**
1.1 ± 0.2	3.5 ± 2.3	48.5 ± 8.5*	0.8 ± 0.3	1 ± 0.8	12.7 ± 7.8§	0.9 ± 0.3	2.1 ± 1.1	21.2 ± 9.6*

* *P* < 0.0001

§ *P* < 0.009

By western blotting, we found low expression of LIGHT in human myeloma cell lines (HMCLs - *i.e.* H929, RPMI-8226, U266) as well as in CD138^+^ myeloma-cells, isolated from MM-bone disease patients. In these cells, by flow cytometry, we detected LIGHT expression at a percentage ranging from 2 to 5 (data not shown). By immunohistochemistry, we demonstrated strong expression of LIGHT in BM biopsy samples from MM-bone disease patients (Figure 1D). We did not find statistically significant difference in LIGHT serum levels among patients with MM-bone disease (207.71 ± 26.53 pg/ml) or symptomatic MM without bone disease (179.84 ± 20.48 pg/ml) as well as in the other samples from patients with sMM (237 ± 89 pg/ml), MGUS (183 ± 20.58 pg/ml), non-neoplastic disease (199 ± 21.2 pg/ml) and healthy-donors (189.84 ± 20.83 pg/ml).

### Anti-LIGHT monoclonal antibody affects osteoclast formation in cultures of PBMCs and BMMNCs from MM-bone disease patients

**Figure 2:**
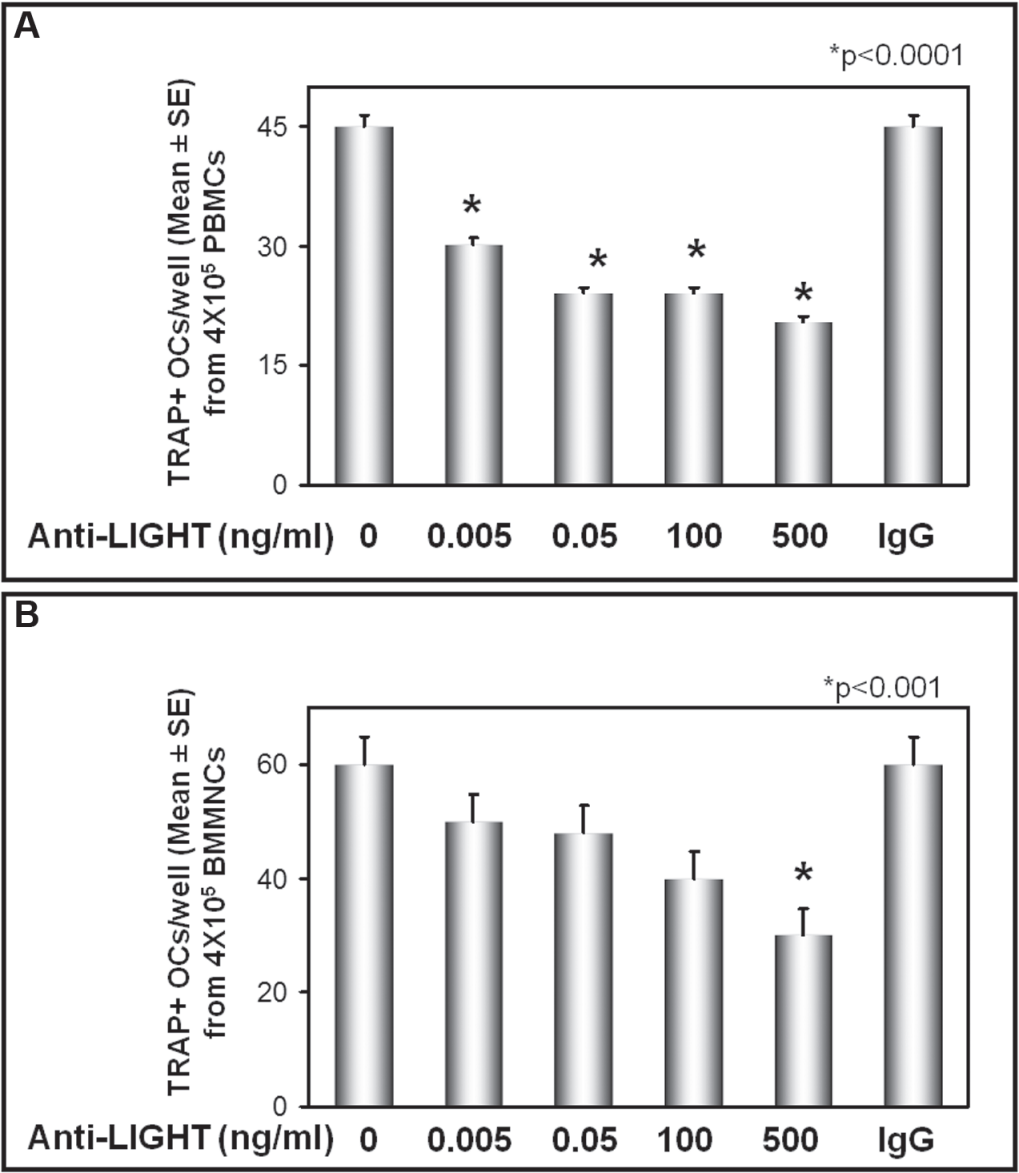
Anti-LIGHT mAb inhibits the osteoclast formation in cultures from MM-bone disease Multinucleated and TRAP+ cells, differentiated from peripheral blood mononuclear cell (PBMC) **(A)** and bone marrow mononuclear cells (BMMNC) **(B)** cultures of MM-bone disease patients, were evaluated after 21 days of culture in the presence of anti-LIGHT mAb or control anti-IgG mAb. The anti-LIGHT mAb culture treatment resulted in a dose-dependent inhibition of osteoclastogenesis, which was not affected by the control anti-IgG mAb. The number of multinucleated and TRAP+ cells, identified as OCs, are represented in the graphs as mean ± SE of all experiments performed in each patient's sample.

In culture media of PBMCs and BM mononuclear cells (BMMNCs) from MM-bone disease patients, we found higher LIGHT levels than in those from controls (1939 ± 220 pg/ml vs 74.7 ± 30 pg/ml, *p* < 0.001; 1750 ± 352 pg/ml vs 66.2 ± 44 pg/ml, *p* < 0.01, respectively). The culture treatment with sequential escalating doses, ranging from 0.005 to 500 ng/ml, of anti-LIGHT monoclonal antibody (mAb) induced a dose-dependent inhibition of osteoclastogenesis, quite different in cultures derived from PBMCs and BMMNCs, respectively. Indeed in PBMC cultures, 30% inhibition of osteoclastogenesis was induced by the lowest dose (0.005 ng/ml) of the anti-LIGHT mAb, increasing up to 50% at the highest dose of the mAb (500 ng/ml) (Figure 2A). In BMMNC cultures, the highest dose (500 ng/ml) of the mAb was instead required to observe a 50% reduction of osteoclastogenesis rate (Figure 2B). Since spontaneous osteoclastogenesis *in vitro* occurred only in MM-bone disease patients, we did not test the effect of anti-LIGHT mAb in PBMCs and BMMNCs of MGUS and sMM patients.

### Anti-LIGHT mAb affects osteoblast differentiation in cultures of BMNCs from MM-bone disease patients

**Figure 3:**
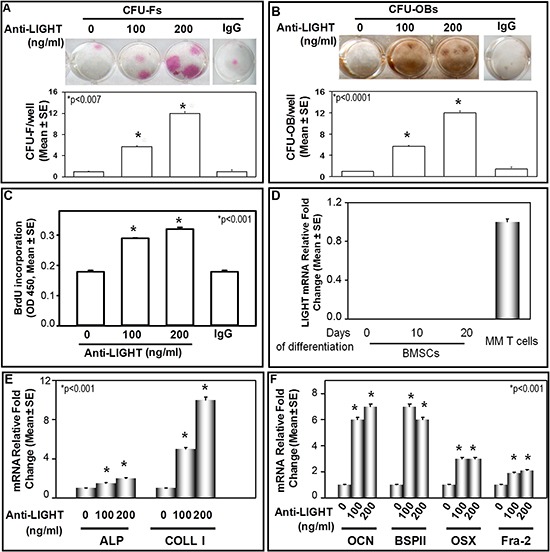
Anti-LIGHT mAb effect on CFU-F and CFU-OB formation in BMNC cultures of MM-bone disease patients **(A)** CFU-F and **(B)** CFU-OB detected in bone marrow nuclear cell (BMNC) cultures from MM-bone disease patients, in the absence (0) or presence of 100 or 200 ng/ml anti-LIGHT neutralizing mAb. The results were compared to those obtained from untreated BMNC cultures. Anti-LIGHT mAb significantly increased CFU-F (A) and CFU-OB (B) formation in a dose-dependent manner. No effect was detected in the presence of a control anti-IgG mAb. The graphs represent the mean number of CFU-F/well or CFU-OB/well ± SE of 6 independent experiments performed in triplicate. **(C)** 5-bromo-2-deoxyuridine (BrdU) incorporation evaluated in BMNC cultures from MM-bone disease patients, treated or not with 100 and 200 ng/ml anti-LIGHT mAb, which positively affected cell proliferation. The graph represents BrdU incorporation evaluated in 6 experiments performed in quadruplicate. **(D)** Purified bone marrow stromal cells (BMSCs) cultured in osteogenic medium for 0, 10 and 20 days did not express LIGHT mRNA. The positive control was represented by T-cells from MM-bone disease patients. **(E)** Anti-LIGHT mAb increased the expression at mRNA level of Alkaline Phosphatase (ALP) and Collagen-I (COLL-I). **(F)** Anti-LIGHT mAb increased the expression at mRNA level of osteocalcin (OCN), bone sialoprotein II (BSP II), Osterix (OSX) and Fra-2. The graphs show 6 real-time PCR experiments performed in triplicate.

To investigate whether LIGHT could be implicated in the impaired OB differentiation occurring in MM-bone disease, we performed long-term cultures (allowing cell to cell contacts) of patients' bone marrow nuclear cells (BMNCs), in the absence or presence of the anti-LIGHT mAb (100 or 200 ng/ml). CFU-F and CFU-OB formation was referred to the early and the late phase of OB differentiation, respectively. In the absence of anti-LIGHT mAb, the formation rate of both the CFU-F and CFU-OB was low (Figure 3 A-B). Otherwise, in the presence of anti-LIGHT mAb at both the concentrations, a dose-dependent increase of CFU-F and CFU-OB formation occurred (Figure 3 A, B). The anti-LIGHT mAb seems to exert the above effects through the induction of BM stromal fraction proliferation, as shown with the 5′-Bromo-2′-deoxyuridine (BrdU) test. Indeed, there was a dose-dependent increase of BrdU incorporation by the BMNCs, cultured in the presence of 100 or 200 ng/ml anti-LIGHT mAb (Figure 3C). Thereafter by flow cytometry, we identified the CD45+ cells as the source of LIGHT in CFU-F and CFU-OB cultures from MM-bone disease patients (data not shown). Additionally, the purified BM stromal cell fraction (BMSCs), cultured in osteogenic medium, did not express LIGHT mRNA in contrast with the positive control, consisting of LIGHT expressing T-cells from MM-bone disease patients, as shown in Figure 3D. By real-time PCR in CFU-F cultures from those patients, we found a low expression of ALP and COLL-I, strongly increased after treatment with 100ng/ml or 200ng/ml anti-LIGHT mAb (Figure 3E). Further in CFU-OB untreated cultures, we detected a low expression of BSPII and OCN (matrix glycoproteins typically expressed during the late phase of OB differentiation) as well as of Fra-2 and OSX (transcription factors), as represented in Figure 3F. In the presence of 100 or 200ng/ml anti-LIGHT mAb, we detected significantly increased mRNA levels of the above molecules. The control anti-IgG mAb did not affect such mRNA expression (data not shown).

### LIGHT induces osteoclast differentiation from healthy-donor PBMCs and purified CD14+ monocytes

**Figure 4:**
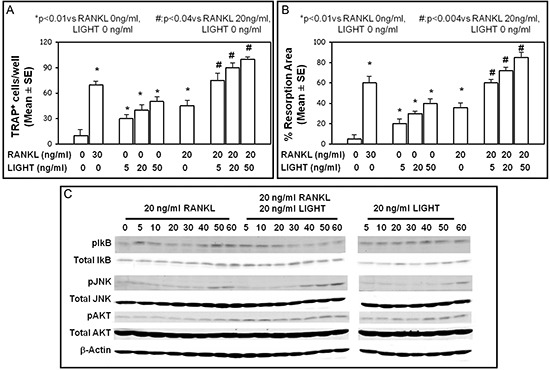
LIGHT pro-osteoclastogenic effect LIGHT treatment of cultures of PBMCs from healthy-donors resulted in dose-dependent increase of OC formation **(A)** and resorption activity **(B)**. These effects were more evident after culture treatment with both RANKL and LIGHT. TRAP+ OC number (A), and resorption area percentage (B) were assessed, and the results represented the mean ± SE of 12 independent experiments. **(C)** Western blot analysis of phosphorylated AKT, JNK, IKB in pre-osteoclasts from healthy-donors incubated with RANKL, RANKL + LIGHT, or LIGHT over a time course (0 to 60 min).

We investigated the effect of LIGHT on OC differentiation in cultures of unfractionated PBMCs or purified CD14+ monocytes from healthy-donors. In these cultures, the monocyte precursors differentiate into multinucleated TRAP+ OCs within 18–21 days, only in the presence of MCSF (25 ng/ml) and RANKL (30 ng/ml). In PBMC cultures treated with MCSF and LIGHT at increasing concentrations (5, 20 or 50 ng/ml), a dose-dependent formation of multinucleated and TRAP+ cells was also seen. In the presence of LIGHT, however, the formation of a lower number of OCs than in RANKL (30 ng/ml) treated cultures appeared. Moreover, in PBMC cultures treated with both LIGHT (at the concentrations of 5, 20 or 50 ng/ml) and RANKL (at a sub-optimal dose of 20 ng/ml), active osteoclastogenesis occurred. Herein, the rate of OC formation was significantly higher than in the presence of either LIGHT or RANKL alone (Figure 4A). The OCs formed in the presence of LIGHT (with or without RANKL) were functional, as demonstrated by their ability in resorbing mineralized matrix. In particular, in the PBMC cultures, OC resorption area increased proportionally to the escalating concentrations of LIGHT; this aspect was more evident in cultures treated with both LIGHT and RANKL, as represented in Figure 4B. The same results, referred to both the number of formed OCs and the resorption area, were obtained in cultures from purified CD14+ monocytes (data not shown).

To further investigate LIGHT effect on OC formation in the presence or absence of RANKL, we analyzed both the intracellular signal and the expression of HVEM and LTβR in pre-osteoclasts treated with RANKL and/or LIGHT. RANKL as well as LIGHT induced the phosphorylation of I#x03BA;Bα (at 5–10 min for RANKL, and longer for LIGHT), JNK (maximum at 50–60 min) and Akt (maximum at 40–60 min) (Figure 4C). Otherwise, the simultaneous treatment with LIGHT and RANKL resulted in early and/or prolonged phosphorylation of Akt (persisting for all the investigated times), IκBα (maximum at 5–20 min) and JNK (maximum at 40–60 min) (Figure 4C). By contrast, the expression of HVEM and LTβR did not significantly change after the pre- osteoclast exposure to RANKL, LIGHT or both (data not shown).

### LIGHT effect on osteoblastogenesis

**Figure 5:**
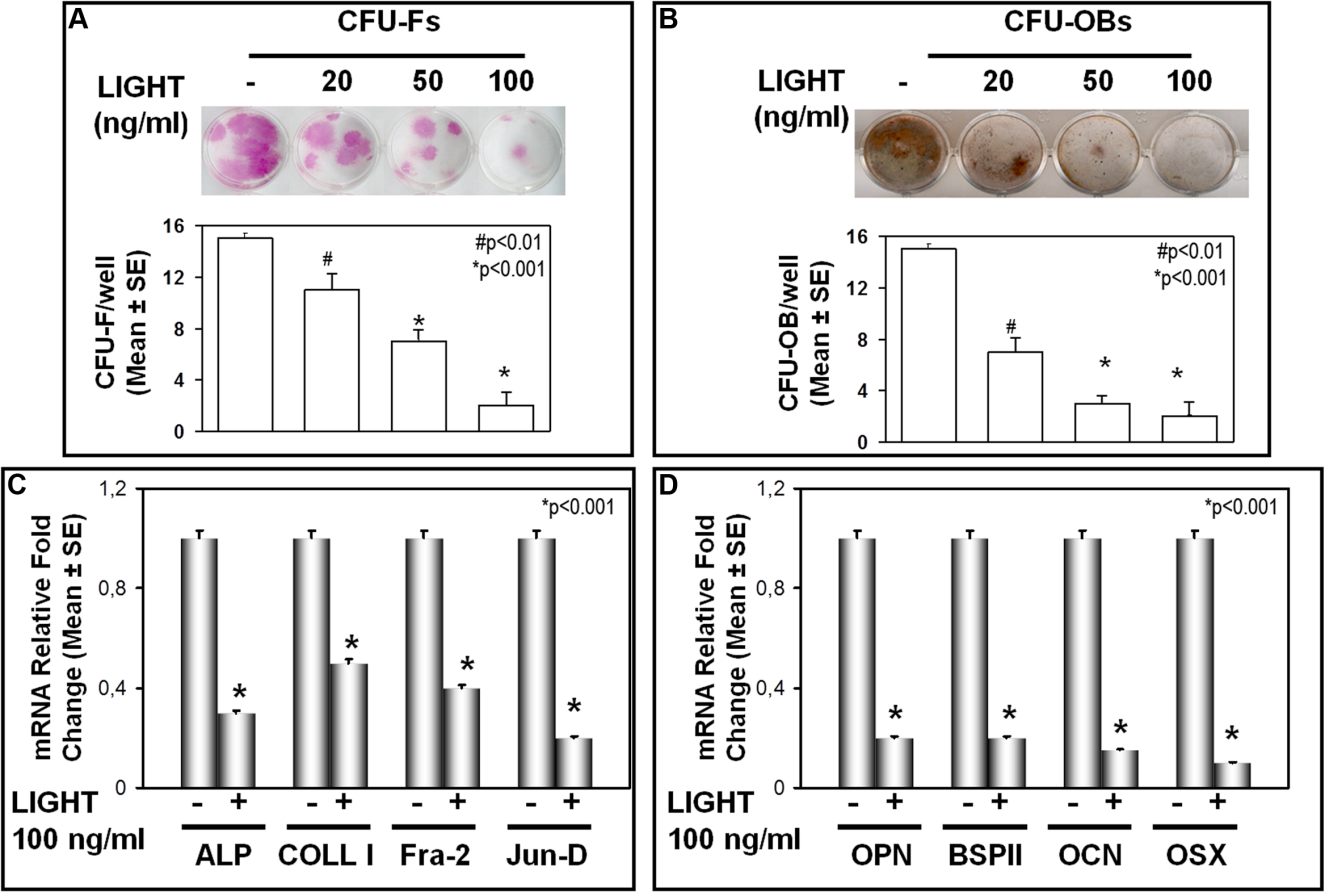
LIGHT effect on CFU-F and CFU-OB formation **(A)** CFU-F and **(B)** CFU-OB formation were detected in BMNC cultures from MGUS and non-neoplastic patients in the absence (0) or presence of 20, 50 or 100 ng/ml LIGHT. The results were compared to those obtained from parallel untreated cultures. LIGHT significantly inhibited both CFU-F (A) and CFU-OB (B) formation in a dose-dependent manner. The graphs represent the mean number of CFU-F/well or CFU-OB/well ± SE of 6 independent experiments performed in triplicate. **(C)** LIGHT inhibited the expression at mRNA level of Alkaline Phosphatase (ALP), Collagen-I (COLL-I), Fra-2 and Jun-D. **(D)** LIGHT significantly inhibited the expression at mRNA level of osteopontin (OPN), bone sialoprotein II (BSP II), osteocalcin (OSC) and Osterix (OSX). The graphs show 6 real-time PCR experiments performed in triplicate.

We investigated the effect of LIGHT on osteoblastogenesis by assessment of CFU-F and CFU-OB formation, occurring in cultures of BMNCs from patients without MM-bone disease (including symptomatic MM, sMM, MGUS, non-neoplastic disease), in the presence or absence of 100 or 200 ng/ml LIGHT. In BMNC cultures, we found that LIGHT treatment strongly inhibited both CFU-F (Figure 5A) and CFU-OB (Figure 5B) formation in a dose-dependent manner. Moreover, we found that LIGHT impaired the expression of osteogenic markers of both early and late phase of OB differentiation. In the early phase, LIGHT particularly inhibited the expression of Alkaline Phosphatase (ALP), and Collagen-I (COLL-I), Fra-2 and Jun-D (Figure 5C); differently in the late phase, it decreased mRNA levels of osteopontin (OPN), bone sialoprotein II (BSP II), osteocalcin (OCN) and Osterix (OSX) (Figure 5D).

**Figure 6:**
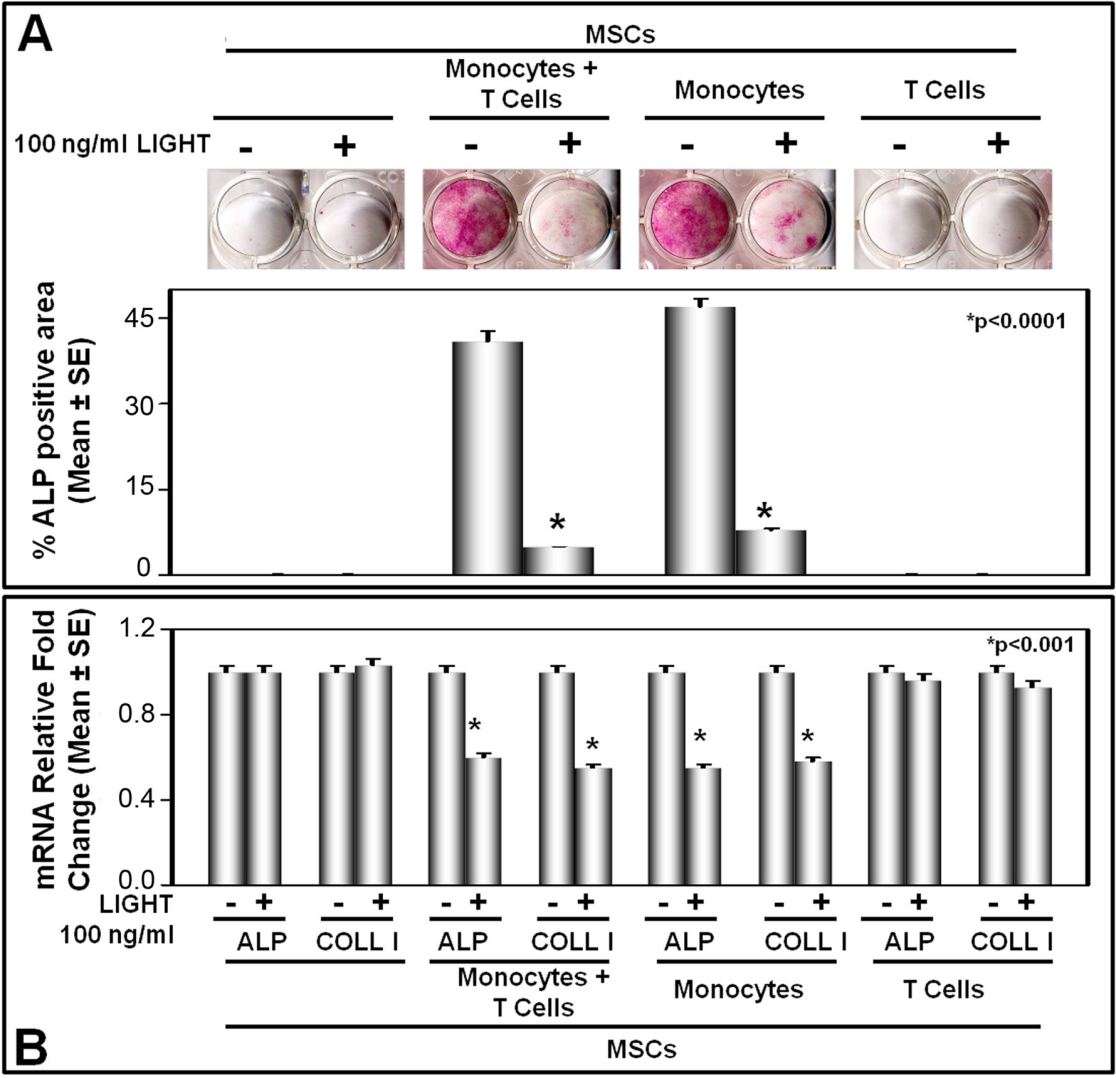

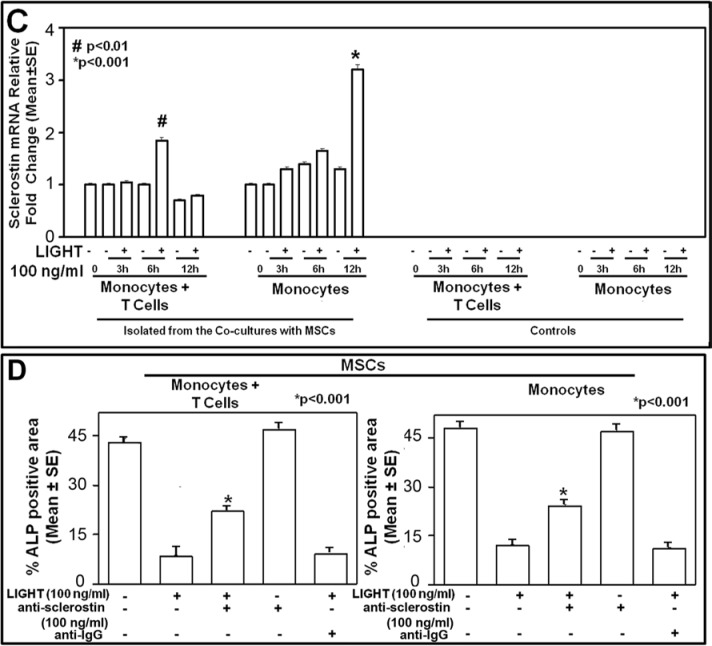
LIGHT effect on osteoblastogenesis in co-cultures of mesenchymal stem cells and monocytes with or without T-cells **(A)** For 8 days in osteogenic medium and in the presence or absence of 100 ng/ml LIGHT, mesenchymal stem cells (MSCs) were cultured alone or co-cultured with monocytes + T-cells, monocytes, or T-cells, respectively. In co-cultures of MSCs with either monocytes + T-cells or monocytes alone, LIGHT significantly inhibited the differentiation of Alkaline Phosphatase positive (ALP+) osteoblasts. The graphs represent the mean percentage of ALP positive area/well ± SE of 5 independent experiments performed in triplicate. **(B)** In the same conditions, LIGHT inhibited the expression, at mRNA level, of Alkaline Phosphatase (ALP) and Collagen-I (COLL-I) in co-cultures of MSCs with monocytes + T-cells, or monocytes alone, respectively. **(C)** In the absence or presence of 100 ng/ml LIGHT over a time course [0 to 12 hours (h)], cultures of monocytes + T-cells or monocytes alone as well as co-cultures of MSCs with monocytes + T-cells, or monocytes alone were performed. Thereby, sclerostin expression was evaluated in the cell extracts from each co-culture consisting of monocytes + T-cells or monocytes alone. The results showed a significant sclerostin up-regulation occurring at 6 hours in the co-coltures of MSCs with monocytes + T-cells, and at 12 hours in those of MSCs with monocytes alone. **(D)** For 8 days in osteogenic medium and in the presence or absence of 100 ng/ml LIGHT and/or 100 ng/ml anti-sclerostin mAb, mesenchymal stem cells (MSCs) were cultured alone and co-cultured with monocytes + T-cells or monocytes, respectively. By treating the above described co-cultures simultaneously with LIGHT and a neutralizing anti-sclerostin mAb, LIGHT inhibitory effect on the differentiation of ALP+ osteoblasts was partially reverted.

These experiments, however, did not indicate whether LIGHT exerts a direct or indirect effect on osteoblastogenesis, because BMNCs include mesenchymal (*i.e.* stromal) cells differentiating into OBs and hematopoietic cells. On the other hand, the latter cells are known to express LIGHT receptors [17–19], and to secrete soluble factors positively affecting CFU-F and CFU-OB survival and proliferation [33]. To address this issue, we performed cultures of either bone marrow stromal cells (BMSCs) or mesenchymal stem cells (MSCs) from dental follicle, which allowed to exclude hematopoietic cell involvement in LIGHT-mediated osteoblastogenesis; both BMSCs and MSCs are indeed lacking in hematopoietic cell fraction. The BMSCs as well as the MSCs were cultured in osteogenic medium and in the presence or absence of LIGHT; MSCs were co-cultured with monocytes and/or T-cells, too. LIGHT resulted to affect osteoblastogenesis neither in the cultures of BMSCs (data not shown) nor in those of MSCs alone (Figure 6A), suggesting that it is not able to exert a direct effect on osteoblastogenesis. Conversely, in the co-cultures of MSCs either with monocytes plus T-cells or with monocytes alone, we detected a significant LIGHT impairment of osteoblastogenesis (Figure 6A). Indeed in LIGHT treated co-cultures, we found significantly reduced ALP positive surface/well (Figure 6A) as well as lower levels of ALP and COLL-I mRNA than in the untreated co-cultures (Figure 6B). Albeit slightly, LIGHT inhibitory effect on osteoblastogenesis was more evident in the co-cultures of MSCs with monocytes plus T-cells than in those with monocytes alone. Finally, no LIGHT effect on osteoblastogenesis was detected in the co-cultures between MSCs and T-cells (Figure 6A).

### High Sclerostin levels were associated to LIGHT inhibitory effect on osteoblastogenesis

In order to investigate the mechanism of LIGHT inhibitory effect on osteoblastogenesis, we treated the co-cultures of MSCs with monocytes alone or monocytes plus T-cells with 100 ng/ml LIGHT for 3, 6 and 12 hours, after over-night MSC adhesion. At each time, cell extracts from isolated monocytes, and from monocytes plus T-cells were obtained after purity assessment (99% CD45^+^) by flow cytometry. The above cell extracts were then evaluated for the expression of genes involved in osteoblastogenesis inhibition such as TNFα, DKK1 and sclerostin. Among these, statistically significant results were obtained only for sclerostin, which is known to be produced by osteocytes [34]. After 6 h LIGHT treatment, increased levels of sclerostin mRNA were detected in the monocyte plus T-cell extracts; otherwise in the monocyte extracts, sclerostin expression increased to a statistically significant level after 12 h LIGHT treatment, as depicted in Figure 6C. Therefore, the effect of LIGHT on sclerostin expression was stronger in the RNA extracts from monocytes than in those from monocytes plus T-cells. In the cell extracts from the parallel cultures without MSCs, no sclerostin mRNA expression was instead detected (Figure 6C). Interestingly, the treatment of the above co-cultures with both LIGHT and a neutralizing anti-sclerostin mAb, partially reverted the sclerostin inhibitory effect on osteoblastogenesis (Figure 6D).

## DISCUSSION

The results of the present study highlighted the high expression of LIGHT in PB and BM samples from the large majority of MM-bone disease patients at diagnosis, in whom LIGHT was demonstrated to be involved in both increased osteoclastogenesis and decreased osteoblastogenesis.

Firstly, we detected high LIGHT expression in CD14+ monocytes, CD8+ T-cells and CD16+ neutrophils from PB and BM of newly diagnosed patients with MM-bone disease. Conversely, we found a low or undetectable expression of LIGHT in the other samples from patients affected by symptomatic MM without bone disease, sMM, MGUS, non-neoplastic disease and in healthy-donors.

In the literature, LIGHT was reported in rheumatoid arthritis erosive bone-disease as expressed by synovial CD4+ T-cells [35] as well as PB and synovial fluid CD14+ monocytes and CD20+ B-cells [36].

Secondly, by means of an *in vitro* osteoclastogenesis model consisting of PBMC and BMMNC cultures from MM-bone disease patients, we demonstrated that LIGHT exerts a pro-osteoclastogenic effect. Indeed, the neutralizing anti-LIGHT mAb induced a dose-dependent inhibition of osteoclastogenesis spontaneously occurring in both the above cultures derived from MM-bone disease patients. We observed, however, that in PBMC cultures lower doses of the mAb inhibited OC formation, compared with those required to obtain the same effect in the parallel BMMNC cultures. Consistently with the literature including our previous reports [2, 12, 14, 37], these results support the involvement of various cytokines, figuring out the occurrence of alternate or additional pathways concerning OC formation and activity. Additionally, it must be also considered the greater complexity of the cultures derived from BM compared to those from PB [37, 38].

Furthermore, we investigated LIGHT pro-osteoclastogenic effect using cultures of PBMCs or CD14+ monocytes from the healthy-donors, where LIGHT dose-dependently increased the OC formation rate. This finding was amplified in the presence of a sub-optimal concentration of RANKL, indicating that LIGHT pro-osteoclastogenic effect occurs both in a RANKL-dependent and -independent way, as also reported in the literature [31, 32]. Conversely, other Authors did not describe a LIGHT pro-osteoclastogenic effect from CD14+ monocyte precursors [30]; these conflicting results might be explained by different culture conditions, such as the number of monocytes plated by them, which was lower than ours [30]. In addition, we found that LIGHT pro-osteoclastogenic effect is related to phosphorylation of the intracellular signaling, typically activated by RANKL during osteoclastogenesis and including IkB, AKT and JNK pathways [39, 40]. However, other Authors [32] described the phosphorylation of both IkB and AKT, following LIGHT stimulation of RAW264 and HL60, and a low rate of phosphorylation of JNK in HL60; both these murine monocyte cell lines are capable of differentiating into OCs under appropriate conditions [31, 41]. The absence or the low rate of JNK phosphorylation might be related to the use of cell lines rather than primary human cells [32]. We detected early and sustained phosphorylation of IkB, AKT and JNK in the cultures treated with both LIGHT and RANKL, whose synergic pro-osteoclastogenic effect resulted herein highlighted. It seems to resemble a mechanism possibly underlying the *in vivo* bone destruction occurring in MM-bone disease, where RANKL is known to play a pivotal role [2, 12, 13, 42].

Thirdly, the results of the present study showed for the first time that LIGHT also affects OB differentiation in BMNC cultures derived from MM-bone disease patients; in which, the osteoblastogenesis indeed resulted significantly improved by the addition of anti-LIGHT mAb. Moreover in BMNC cultures from patients without bone disease, we demonstrated that the addition of LIGHT inhibited osteoblastogenesis. Since BMNCs include mesenchymal cell fraction and CD45+ cells, we could not argue whether LIGHT induces the osteoblastogenesis impairment through a direct or indirect effect. Thus, we carried on with our study evaluating OB differentiation from BMSCs of patients with or without bone disease, in the presence or absence of LIGHT. In addition, we assessed the effects of LIGHT on MSCs isolated from dental follicle, cultured alone or co-cultured with monocytes and/or T-cells. This model was suitable for our purposes since MSCs are known to be immunologically privileged cells [43]. Since no effect of LIGHT on OB differentiation was detected either in cultures of BMSCs or in those of MSCs alone, the event of a LIGHT direct effect could be excluded. On the contrary, the results we detected in the co-cultures of MSCs with monocytes alone and/or T-cells pointed out a LIGHT indirect effect, to some extent occurring through the release of sclerostin by monocytes. Sclerostin is indeed a key inhibitor of OB differentiation, known to be produced by osteocytes [34], whereas there are no literature data concerning its expression by monocytes. Some Authors, however, described sclerostin expression within the hematopoietic islands of embryo livers and by OCs, which are known to be derived from the fusion of monocyte precursors [44, 45]. Therefore, we could argue that sclerostin may be expressed by monocytes under particular conditions. There are numerous literature reports on monocyte involvement in osteoblastogenesis [46–48]; in particular, bone resident macrophages have been recognized as critical regulators of bone homeostasis and repair in a murine tibia injury model [48]. The increase of cytokines such as IL-1β, IL-6, IL-10 and TNFα was described as following the MSC-monocyte contact [49–53]; no role was, however, recognized to these cytokines in the enhanced monocyte-induced osteogenesis. In studies using MG-63 osteoblastic cell line or calvarial OBs, IL-6 has been described as involved in the direct promotion of osteogenic differentiation [54, 55]. More recently, monocytes have been reported as regulating the osteogenic differentiation of MSCs through cell-contact mechanisms involving Oncostatin-M in monocytes, and STAT-3 signaling in MSCs [56].

In conclusion, the results of the present study show for the first time high expression of LIGHT by monocytes, T-cells and neutrophils from symptomatic MM patients with bone disease. Further, our results show that LIGHT seems to be implicated in the development of MM-bone disease through a direct pro-osteoclastogenic effect and an indirect induction of OB suppression. Based on these findings, LIGHT provides a potential target for novel therapeutic strategies.

## PATIENTS AND METHODS

### Patients

PB, bone marrow (BM) aspirates and biopsies were obtained from 80 patients newly diagnosed as having symptomatic MM (58 of them with, and 22 without related bone disease), 16 with smoldering MM (sMM), and 35 with monoclonal gammopathy of undetermined significance (MGUS). The control samples included PB and BM from 10 patients with non-neoplastic disease without skeletal involvement, and PB from 30 healthy volunteer blood donors (healthy-donors), age and sex matching with the patients.

The patient characteristics are reported in Table 2. All patients underwent skeletal X-ray, and some of them also required magnetic resonance imaging or computerized tomography to assess symptomatic bone sites, pathological fractures, cord compression or tumour mass. Patient diagnoses were performed according to the International Myeloma Working Group's (IMWG) criteria [1, 57], and symptomatic MM was also classified according to the International Staging System (ISS) [58]. Patients and controls gave their informed consent to the study performed according to the Declaration of Helsinki, and approved by Bari University Hospital Ethical Committee.

**Table 2:** Characteristics of patients and controls

Parameters	Symptomatic MM at diagnosis		Smoldering MM	MGUS	Controls	
	*With bone disease*	*Without bone disease*			*Patients with non-neoplastic disease*	*Healthy-donors*
Number of subjects	58	22	16	35	10	30
Gender (M/F)	30/28	12/10	9/7	18/17	6/4	18/12
Median age (range)	69 (60–85)	64 (54–84)	55 (31–83)	60 (29–83)	63(50–73)	62 (26–72)
Monoclonal Component:						
IgG/IgA/BJ/IgD/NS	47-8-2-1-0	18-3-1-0-0		25-10-0-0-0	-	-
ISS-stage	ISS-1: 20	ISS-1: 11				
	ISS-2: 18	ISS-2: 6				
	ISS-3: 20	ISS-3: 5				
Hb < 10 g/dL	12	5		-		-
Creatinine ≥ 2 mg/dl	5	4		-		-
Albumin < 3.5 g/dL	15	2		-		-
β2-M > 3 mg/L	8	2		-		-
β2-M > 6 mg/L	7			-		-
LDH > 240 U/L	4	1		-		-
Calcium > 10 mg/dl	-			-		-

Abbreviation: MM, multiple myeloma; MGUS, monoclonal gammopathy of undetermined significance; M, male; F, female; Ig, immunoglobulin; Hb, hemoglobin; CRP, C-reactive protein; LDH, lactate deydrogenase; ISS, International Staging System; NS, non secretory.

### Cells and cell cultures

#### Human myeloma cell lines (HMCLs) and CD138+ cells

HMCLs (H929, RPMI-8226, U266) were cultured in RPMI-1640 medium supplemented with 10% fetal bovine serum (FBS; Life Technologies, Milan, Italy). Plasma cells, identified as CD138+ cells, were isolated from BM aspirates [8]. RNA or proteins were extracted from HMCLs and fresh CD138+ cells to evaluate LIGHT expression.

#### Bone marrow and peripheral blood cells

Buffy coat BM nuclear cells (BMNCs) and PB mononuclear cells (PBMCs), isolated by Histopaque 1077 density gradient (Sigma, St Louis, MO), were plated. Thereby, BM stromal cells (BMSCs) and BM mononuclear cells (BMMNCs) were obtained from BMNC adherent and non-adherent fractions, respectively. BMMNC and PBMC cultures were performed to investigate osteoclastogenesis, whereas BMNCs and BMSCs were cultured to investigate osteoblastogenesis.

#### Mesenchymal stem cells (MSCs), bone marrow and peripheral blood cells

MSCs, isolated as previously described [59], were used for osteoblastogenesis experiments.

#### CD14+ monocyte, CD2+ T-cell and neutrophil isolation

From PBMCs and BMNCs, CD14+ and CD2+ cells were purified by immunomagnetic selection (Miltenyi Biotec GmbH, Bergisch Gladbach, Germany), according to the manufacturer's instruction. Neutrophils were isolated as previously described [60]. Only samples with a purity >98%, checked by flow cytometry, were considered. RNA and proteins were extracted from purified CD14+ monocytes, CD2+ T-cells and neutrophils to evaluate LIGHT expression. CD14+ monocytes were also used in osteoclastogenesis experiments.

#### Osteoclastogenesis

To investigate LIGHT-dependent OC formation, BMMNCs and PBMCs from MM-bone disease patients were plated at 1.5 × 10^6^ cell/cm^2^ in α-Minimal Essential Medium (α-MEM, Life Technologies), supplemented with 10% FBS and cultured in the absence or presence of anti-LIGHT mAb (0.005 to 500 ng/ml; R&D Systems Inc., Minneapolis, MN) or control anti-immunoglobulin G (IgG)-Ab. PBMCs and purified CD14+ cells from healthy-donors were cultured in the presence or absence of 25 ng/ml recombinant human Macrophage Colony Stimulating Factor (rh-MCSF) *plus* either 30 ng/ml rh-RANKL or rh-LIGHT (range 0-50 ng/ml) (all from R&D Systems). For some experiments, PBMC and CD14+ cell cultures were treated with MCSF (25 ng/ml), RANKL (20 ng/ml), and/or LIGHT (range 5-50 ng/ml). Mature OCs were identified as Tartrate-Resistant Acid Phosphatase (Sigma) positive (TRAP+) cells containing 3 or more nuclei. OC resorbing activity was assessed by plating the cells on Millennium multiwell slides (Millennium Biologix, Kingston, ON, Canada).

To study the phophorylation of IkB, JNK and AKT, healthy-donor PBMCs were cultured for 10 days in the presence of MCSF to obtain pre-osteoclasts, that after overnight starvation were incubated with RANKL, RANKL + LIGHT, or LIGHT over a time course (0 to 60 min).

#### Osteoblastogenesis

BMNCs were plated at 4 × 10^5^/cm^2^ in osteogenic differentiating medium [α-MEM supplemented with 10% FBS, 50 μg/mL ascorbic acid, 10^−8^ M dexamethasone and 10 mM beta-glycerophosphate (Sigma)]. BMNCs from patients without bone disease were cultured in the presence or absence of rh-LIGHT (range 20-100 ng/ml). Conversely, BMNCs from MM-bone disease patients were cultured in the absence or presence of 100 and 200 ng/ml neutralizing anti-LIGHT mAb or control anti-IgG Ab. After 21 days, colony forming unit-fibroblast (CFU-F) formation was assessed with alkaline phosphatase (ALP, Sigma) staining. After 30 days, in parallel cultures CFU-OB formation was assessed with Von Kossa staining [8]. The mRNA expression of ALP, collagen-I (COLL-I), Fra-2 and JunD was analyzed in CFU-F cultures, and that of osteopontin (OPN), Osterix (OSX), bone sialoprotein II (BSP II) and osteocalcin (OCN) in CFU-OB cultures.

BMSCs, cultured in α-MEM with 10% FBS, after reaching confluence, were seeded in 6-well plate at 100000/well and cultured in osteogenic medium for 0, 10 and 20 days. At each time, RNA was extracted to evaluate LIGHT expression.

MSCs, seeded in 24-well-plate at 400/well, were cultured alone for 8 days in osteogenic differentiating medium, in the absence or presence of 100 ng/ml LIGHT and/or anti-sclerostin mAb (R&D Systems). In the same conditions, MSCs were also co-cultured with monocytes alone, T-cells alone or monocytes plus T-cells at 750000 cell/well, respectively. At the end of culture period, ALP staining or mRNA extraction were performed to evaluate ALP and COLL-I expression.

#### Immunohistochemistry

BM biopsies were fixed in 4% neutral buffered formalin solution, decalcified, paraffin-embedded, and cut into 5 μm slices. After standard antigen retrieval procedures, the sections were incubated overnight at 4°C with primary mouse anti-human LIGHT (Abcam, Cambridge Science Park). The reaction was revealed with Dako EnVision^TM^ FLEX+ detection system (Dako Italia S.p.A. Milan, Italy).

#### Flow cytometry analysis

Freshly BM and PB samples were stained with suitable conjugated antibody: PE-LIGHT (R&D Systems), CD45-APC-Cy7, FITC-CD8, FITC-CD4, Pe-Cy-5-CD14 and FITC-CD16 (Becton Dickinson, Milan, Italy). Flow cytometry analysis was performed on a FACSCantoTM II or BD Accuri™ C6 flow cytometer (Becton Dickinson Immunocytometry System, Mountain View, CA, USA). Positivity area was determined using an isotype-matched mAb, and a total of 2000 events for each cell sub-population was acquired.

#### RNA isolation and real-time polymerase chain reaction (PCR) amplification

RNA extraction and reverse-transcription as well as Real-Time PCR amplification were performed, as previously described [12]. The appropriate primer pairs were listed in Table 3.

**Table 3:** Sense and antisense primer sequences

Gene	Sense primer	Antisense primer	Accession number
LIGHT	5′ CAGTGTTTGTGGTGGATGG 3′	5′ GGGTTGACCTCGTGAGAC	NM_003807.3
ALP	5′ CGCACGGAACTCCTGACC 3′	5′ GCCACCACCACCATCTCG 3′	NM_000478.4
COLL 1	5′ CGTGGCAGTGATGGAAGTG 3′	5′ AGCAGGACCAGCGTTACC 3′	NM_000089.3
OCN	5′ ACACTCCTCGCCCTATTG 3′	5′ CAGCCATTGATACAGGTAGC 3′	NM_199173.4
BSP II	5′ CTGCTACAACACTGGGCTATG 3′	5′ TTCCTTCCTCTTCCTCCTCTTC 3′	NM_004967.3
OSX	5′ GCAAGGTGTATGGCAAGG 3′	5′ CATCCGAACGAGTGAACC 3′	NM_001173467.1
Fra-2	5′ GAACCTCGTCTTCACCTATCC 3′	5′ CCGCTGCTACTGCTTCTG 3′	NM_005253.3
Jun-D	5′ CTCATCATCCAGTCCAAC 3′	5′ GTTCTGCTTGTGTAAATCC 3′	NM_001286968.1
OPN	5′ CTGATGAATCTGATGAACTGGTC 3′	5′ GTGATGTCCTCGTCTGTAGC 3′	NM_001251830.1
Sclerostin	5′ CAGCCTTCCGTGTAGTGG 3′	5′ TTCATGGTCTTGTTGTTCTCC 3′	NM_025237.2
β-Actin	5′ AATCGTGCGTGACATTAAG 3′	5′ GAAGGAAGGCTGGAAGAG 3′	NM_001101.3

Abbreviation: ALP, Alkaline Phosphatase; COLL I, Collagen Type I; OCN, Osteocalcin; BSP II, Bone Sialoprotein; OSX, Osterix; OPN, Osteopontin.

#### Western blot analysis

Cell protein extracts were analyzed by western blotting, as previously described [12]. The following primary antibodies were used: anti-LIGHT and anti-β-actin (Santa Cruz Biotechnology, Santa Cruz, CA), anti-pAKT, anti-pIkB, anti-pJNK, anti-total AKT, anti-IkB, anti-total-ERK and anti-total JNK (Cell Signaling, San Diego, CA, USA).

#### Cell proliferation assay

After 10 days of culture, the anti-LIGHT effect on BMNC proliferation was evaluated with BrdU incorporation by using a cell proliferation enzyme-linked immunosorbent assay (ELISA) kit (Roche Diagnostics, Mannheim, Germany) according to the manufacturer's instructions.

#### ELISA

Patient and control sera as well as media from BMNC, PBMC and BMMNC cultures, were evaluated for LIGHT by ELISA (R&D Systems) according to manufacturer's instructions. The results were expressed as mean ± standard error (SE).

#### Statistical analyses

Statistical analyses were performed by ANOVA or Student's t-test with the Statistical Package for the Social Sciences (spssx/pc) software (SPSS, Chicago, IL, USA). The results were considered statistically significant for *p* < 0.05.
